# Association between Genetic Subgroups of Pancreatic Ductal Adenocarcinoma Defined by High Density 500 K SNP-Arrays and Tumor Histopathology

**DOI:** 10.1371/journal.pone.0022315

**Published:** 2011-07-21

**Authors:** María Laura Gutiérrez, Luís Muñoz-Bellvis, María del Mar Abad, Oscar Bengoechea, María González-González, Alberto Orfao, José María Sayagués

**Affiliations:** 1 Servicio General de Citometría, Departamento de Medicina and Centro de Investigación del Cáncer (IBMCC-CSIC/USAL), Universidad de Salamanca, Salamanca, Spain; 2 Unidad de Cirugía Hepatobiliopancreática y Trasplante de Páncreas, Departamento de Cirugía, Hospital Universitario de Salamanca, Salamanca, Spain; 3 Departamento de Patología, Hospital Universitario de Salamanca, Salamanca, Spain; Tor Vergata University of Rome, Italy

## Abstract

The specific genes and genetic pathways associated with pancreatic ductal adenocarcinoma are still largely unknown partially due to the low resolution of the techniques applied so far to their study. Here we used high-density 500 K single nucleotide polymorphism (SNP)-arrays to define those chromosomal regions which most commonly harbour copy number (CN) alterations and loss of heterozygozity (LOH) in a series of 20 PDAC tumors and we correlated the corresponding genetic profiles with the most relevant clinical and histopathological features of the disease. Overall our results showed that primary PDAC frequently display (>70%) extensive gains of chromosomes 1q, 7q, 8q and 20q, together with losses of chromosomes 1p, 9p, 12q, 17p and 18q, such chromosomal regions harboring multiple cancer- and PDAC-associated genes. Interestingly, these alterations clustered into two distinct genetic profiles characterized by gains of the 2q14.2, 3q22.1, 5q32, 10q26.13, 10q26.3, 11q13.1, 11q13.3, 11q13.4, 16q24.1, 16q24.3, 22q13.1, 22q13.31 and 22q13.32 chromosomal regions (group 1; n = 9) versus gains at 1q21.1 and losses of the 1p36.11, 6q25.2, 9p22.1, 9p24.3, 17p13.3 and Xp22.33 chromosomal regions (group 2; n = 11). From the clinical and histopathological point of view, group 1 cases were associated with smaller and well/moderately-differentiated grade I/II PDAC tumors, whereas and group 2 PDAC displayed a larger size and they mainly consisted of poorly-differentiated grade III carcinomas. These findings confirm the cytogenetic complexity and heterogenity of PDAC and provide evidence for the association between tumor cytogenetics and its histopathological features. In addition, we also show that the altered regions identified harbor multiple cancer associate genes that deserve further investigation to determine their relevance in the pathogenesis of PDAC.

## Introduction

Pancreatic ductal adenocarcinoma (PDAC) is a fatal disease with a 5-year mortality rate of almost 100%. As in other types of cancer, understanding of the molecular mechanisms involved in tumor development and progression is a prerequisite to improve early diagnosis and therapy. Usage of a wide battery of techniques such *in situ* fluorescence hybridization (FISH), comparative genomic hybridization (CGH) and array-CGH (aCGH), has allowed identification of multiple specific recurrently altered chromosomal areas in PDAC tumors; most frequently reported alterations include losses of chromosomes 8p, 9p, 17p and 18q, together with gains of chromosomes 3q, 8q and 20q [1]–[4]. However, the identification of the specific genes targeted by such abnormalities has proven difficult with these approaches, partially due to the fact that these techniques have a relatively limited resolution. In fact, the highest resolution of such approaches applied so far to the study of PDAC are based on aCGH[5], [6] which has proven to be still relatively limited in resolution for detailed characterization of small regions carrying genetic changes and the identification of the involved genes.

The development of wide-genome approaches such as high-density single nucleotide polymorphism (SNP)-arrays, has further improved the sensitivity of aCGH and provided the opportunity for large scale genotyping with a more accurate definition of the magnitude of the abnormalities detected, through the identification of copy number variation (CNV) and loss of heterozigosity (LOH) for hundreds of thousands of SNPs[7]. This allows highly precise mapping of those genetic changes occurring across the entire genome in a major fraction of all tumor cells, providing a promising starting point for the identification of novel candidate genes affected by such genomic alterations and profiles. To the best of our knowledge, only Jones *et al* and Harada *et al* [8], [9] have previously applied the SNP-array technology to primary PDAC samples and none of them has investigated so far the potential association between SNP-array profiles of copy number alterations and tumor histopathology.

In the present study, we applied higher density 500 K SNP arrays with a 2.5 Kb of resolution, to a series of 20 PDAC tumors vs. paired peripheral blood (PB) samples from an identical number of patients who underwent complete tumor resection. Our major goal was to map the most common reccurrent chromosomal alterations present at diagnosis in PDAC tumors and correlate them with the histopathological subtypes of the disease. Overall, the copy number values (CNV) obtained confirm that primary PDAC frequently (>70%) carry extensive gains of chromosomes 1q, 7q, 8q and 20q, together with losses of chromosomes 1p, 9p, 12q, 17p and 18q; these chromosomal regions, contain multiple cancer genes known to be directly related to PDAC disease. Most interestingly, we show for the first time the existence of two major groups of PDAC defined on the basis of the altered SNP-array profiles which showed a close association with tumor histopathology.

## Materials and Methods

### Patients and samples

Tissue specimens were obtained at diagnosis from 20 sporadic PDAC patients (15 males and 5 females) -mean age of 67 years (range: 45 to 84 years)-. All patients underwent surgical tumor resection at the Division of Hepatobiliary and Pancreatic Surgery of the University Hospital of Salamanca (Salamanca, Spain), between October 2003 and October 2008. The study was approved by the local ethics committee of the University Hospital of Salamanca (Salamanca, Spain) and written informed consent was given by each individual prior to entering the study, according to the Helsinki Declaration.

Tumors were diagnosed and classified according to Adsay *et al*.[10] with the following distribution: 5 cases corresponded to well-differentiated/grade I tumors; 7 to moderately-differentiated/grade II, and; 8 to poorly-differentiated/grade III PDAC. Histopathological grade was confirmed in all cases in a second independent evaluation by an experienced pathologist. Most tumors (18/20, 90%) were localized in the head of the pancreas; the remaining two cases were localized in the pancreatic body and body/tail, respectively. Mean tumor size at diagnostic surgery was of 3.0±0.95 cm; 10 cases corresponded to TNM stage IIA tumors and the other 10 to TNM stage IIB. The most relevant clinical and laboratory patient characteristics are summarized in table 1.

**Table 1:** Clinical and biological characteristics of patients with PDAC (n = 20).

Case ID	Gender	Age (years)	CA19.9 serum levels (U/ml)	Localization of primary tumor	Histological grade	Tumor size (cm)	TNM stage		LNR	Perineural Invasion	Vascular Invasion	Type of surgical resection	Genetic subgroup*
1	F	74	>500	Head	I	2	T3N0M0	IIA	0/17	+	-	R0	1
2	M	74	144	Head	I	2.5	T2N1M0	IIB	3/25	+	-	R1	1
3	F	79	177	Head	I	2.5	T2N1M0	IIB	3/28	+	-	R0	1
4	M	64	41.4	Head	I	2.2	T3N1M0	IIB	3/16	+	-	R1	2
5	F	73	377	Head	I	2	T3N0M0	IIA	0/10	+	-	R0	2
6	M	77	>500	Head	II	3.5	T3N1M0	IIB	4/29	+	-	R0	1
7	M	73	<2.5	Head	II	2	T3N1M0	IIB	1/27	-	-	R0	1
8	M	61	3	Head	II	3	T2N0M0	IIA	0/27	+	-	R1	1
9	M	51	>500	Head	II	2.5	T3N1M0	IIB	1/18	+	+	R1	1
10	M	88	89	Head	II	2	T3N0M0	IIA	0/18	-	-	R1	1
11	M	74	45.3	Head	II	3.5	T2N1M0	IIB	5/5	+	-	R1	2
12	M	65	<2.5	Head	II	3	T3N1M0	IIB	2/24	+	-	R0	2
13	M	60	313	Head	III	NA	T3N0M0	IIA	0/0	+	-	R0	2
14	M	74	315	Head	III	4	T3N0M0	IIA	0/37	-	-	R0	2
15	M	56	>500	Head	III	3	T3N0M0	IIA	0/32	+	-	R0	1
16	F	45	>500	Head	III	3.5	T2N1M0	IIB	9/72	+	-	R0	2
17	M	78	176	Head	III	3.5	T2N1M0	IIB	1/27	+	-	R0	2
18	M	62	58	Head	III	2.8	T3N0M0	IIA	0/13	+	-	R0	2
19	F	76	124	Body/tail	III	5.8	T2N1M0	IIB	1/16	-	-	R0	2
20	M	46	<2.5	Body	III	4	T3N0M0	IIA	0/18	+	-	R0	2

M: male; F: female; CA19.9: carbohydrate associated antigen; LNR: lymph node ratio expressed as number of positive lymph nodes from all lymph nodes analyzed; R0: negative microscopic resection margins. R1: positive microscopic resection margins. NA: data not available.

*as defined by hierarchical clustering analysis of CNV obtained by SNP-arrays studies.

Once histopathological diagnosis had been established, part of the tumor sample showing both macroscopical and microscopical infiltration was used to prepare single cell suspensions for iFISH and SNP-array studies. From the paraffin-embedded tissue samples, sections were cut from three different areas representative of the tumoral tissue and placed over poly L-lysine coated slides. All tissues were evaluated after hematoxylin-eosin staining to confirm the presence and determine the quantity of tumor cells infiltrating the material to be studied by SNP-arrays. For SNP-array studies, tumor DNA was extracted from freshly-frozen tumor tissues mirror cut to those used for iFISH analyses, which contained ≥70% tumor cells. In turn, normal DNA was extracted from matched PB leucocytes from the same patient. For both types of samples (tumor tissue and PB leucocytes), DNA was extracted using the QIAamp DNA mini kit (Qiagen, Hilden, Germany) following the manufacturer's instructions.

### SNP-array studies

Paired samples of purified tumoral DNA and normal PB DNA from individual patients were hybridized to two 250 K Affymetrix SNP Mapping arrays each (*Nsp*I and *Sty*I SNP arrays; Affymetrix, Santa Clara, CA, with a median resolution of 2.5 Kb and an average distance between SNPs of 5.8 Kb), using a total of 250 ng of DNA per array, according to the instructions of the manufacturer. Fluorescence signals were detected using the GeneChip Scanner 3000 (Affymetrix) and data stored in CEL files. Analysis of paired tumoral/normal CEL files containing data on the SNP-array results was done using the Genotyping Console software (GTC v2.1, Affymetrix). Genotypes were generated using the BRLMM algorithm included in the GTC software. The mean call rate for individual SNPs was systematically ≥86.5% (median of 98.6%). Copy number (CN) alterations and loss of heterozigocity (LOH) were inferred by a Hidden Markov Model-based algorithm implemented in the GTC software program, using parameter settings recommended by Affymetrix for tumoral/normal paired samples and a minimum physical length of at least 5 consecutive SNPs for putative genetic alterations. “Genetic gains” (CN≥2.5) and “losses” (CN≤1.7) were defined according to GTC working criteria. In turn, “high CN gains” and “homozygous losses” were considered to be present when CN values ≥4 and CN≤0.3 were found, respectively.

At every locus, LOH was assumed to be present when a single allele was detected in tumor DNA from heterozygous individuals at a greater percentage than the other allele; it was further subclassified as either true LOH, when loci at which one of the parental copies of a chromosome was deleted, or as copy neutral LOH (cnLOH), when tumoral DNA displayed two copies of a chromosomal region from one parent in the absence of the allele derived from the other parent. Analysis of LOH was restricted to DNA sequences from autosomal chromosomes.

### Interphase fluorescence in situ hybridization (iFISH) studies

In all cases, iFISH studies were performed on an aliquot of the single cell suspension prepared from the tumor sample. A set of 12 locus-specific FISH probes directed against DNA sequences localized in 11 different human chromosomes and specific for those chromosomal regions more frequently gained or deleted in PDAC, were systematically used to validate the results obtained with the SNP-arrays (Table 2). The methods and procedures used for the iFISH studies have been previously described in detail[11] .

**Table 2:** Correlation between the numerical changes detected by 12 iFISH probes and CN values obtained with the SNP-arrays for the same chromosomal regions in 20 PDAC.

FISH probe	Chromosome localization	iFISH probe length (kb)	N. of SNPs* inside the region identified by the FISH probe	R2 (P-value)
LSI *N-MYC*	2p24	200	38	0.70 (0.001)
LSI *D5S23*	5p15	450	118	0.60 (0.005)
LSI *SEC63*	6q21	275	275	0.76 (<0.001)
LSI *MYB*	6q23	740	88	0.56 (0.01)
LSI *D7S486*	7q31	200	33	0.62 (0.004)
LSI *CMYC*	8q24	600	159	0.79 (<0.001)
LSI *PTEN*	10q23	368	49	0.70 (0.001)
LSI *TEL*	12p13	350	98	0.76 (<0.001)
LSI *LAMP1*	13q34	550	92	0.55 (0.012)
LSI *HER2*	17q11	109	10	0.59 (0.006)
LSI *BCL2*	18q21	750	153	0.64 (0.002)
LSI *AML1*	21q22	500	111	0.60 (0.005)

All probes were purchased from Vysis Inc (Chicago, IL, USA), except for the 6q21 and 12p13, which were obtained from QBIOgene Inc (Amsterdam, The Netherlands).

R^2^: Coefficient of correlation;

*Affymetrix 500 K SNP array plataform; iFISH: Interphase fluorescence *in situ* hybridation.

### Statistical Methods

For all continuous variables, mean values and their standard deviation (SD) and range were calculated using the SPSS software package (SPSS 12.0 Inc, Chicago, IL USA); for dichotomic variables, frequencies were reported. In order to evaluate the statistical significance of differences observed between groups, the Mann-Whitney U and X2 tests were used for continuous and categorical variables, respectively (SPSS). A multivariate stepwise regression analysis (regression, SPSS) was performed to examine the correlation between the chromosomal abnormalities found by iFISH versus SNP-array techniques. Hierarchical clustering analysis was performed to classify cases according to their CN genetic profile by using the Cluster 3.0 software (PAM software; http://www-stat.stanford.edu/~tibs/PAM). Clustering was run using an Euclidean distance metric and the average linkage method. For visualization of dendograms the TreeView software 1.0.4[12] was used. P-values <.01 were considered to be associated with statistical significance.

## Results

### Frequency and type of chromosomal abnormalities detected by SNP-arrays

Identification of chromosomal regions throughout the whole genome of PDACs with CN alterations and LOH showed that those chromosomal regions most frequently lost were 17p12 (15/20 cases; 75%), followed by 1p35, 9p22, 12q23 and 18q21 (14/20 cases; 70%) (Table 3). Similarly, gains were frequently observed at chromosomes 1q21.2 and 8q24.3 (15/20 cases; 75%), followed by chromosomes 7q36 and 20q13 (14/20 cases; 70%). Gains and losses of many other chromosomal regions were identified at lower frequencies (≤65%; Table S1).

**Table 3:** Most frequently (>70%) detected regions of gain, loss and LOH in PDAC genotyped on the Affymetrix 500 K SNP array platform (n = 20).

Chromosomal (Chr) region (bp)		Chromosome band	Length (Kb)	N. of SNPs in the altered region	% of altered cases	Genes involved* (N. of genes)	
**CN Losses**							
chr1:	28,370,650–28,562,758	p35.3	192	16	70%	***EYA3, PTAFR***	(3)
chr9:	14,980,485–15,038,399	p22.3	57.9	10	70%		(1)
chr12:	95,450,296–95,526,343	q23.1	76	16	70%	***NR2C1***	(2)
chr17:	2,981,856–3,082,150	p13.3	100	23	70%		(2)
	9,200,905–9,347,984	p13.1	347	38	70%		(1)
	11,246,600–11,347,984	p12	101	36	75%		(1)
	12,306,804–12,540,753	p12	234	44	75%	***MYOCD***	(2)
	47,053,989–47,085,681	q21.1	31.7	9	70%		(0)
	47,095,478–47,133,926	q21.1	38.5	11	70%		(1)
chr18:	50,371,940–50,528,205	q21.2	156	29	70%	***DCC***	(1)
	53,612,861–53,710,464	q21.31	97.6	26	70%		(1)
**CN Gains**							
chr1:	147,306,690–147,521,567	q21.2	214.9	8	75%		(6)
	150,306,335–150,329,391	q21.3	23	5	70%	***PRPF3***	(1)
	150,355,573–150,496,340	q21.3	140.8	30	70%	*TARS2, **ECM1***	(5)
chr7:	154,876,017–154,924,521	q36.3	48.5	18	70%		(1)
	155,386,030–155,490,447	q36.3	104	22	70%		(1)
	157,638,633–157,783,490	q36.3	91	20	70%	***PTPRN2***	(1)
chr8:	142,428,444–142,726,810	q24.3	298	46	70%	***PTP4A3***	(3)
	142,754,129–143,356,971	q24.3	602.8	99	75%		(2)
	143,374,709–143,377,003	q24.3	2.3	5	70%		(1)
	143,564,928–143,854,26	q24.3	289	30	70%	***BAI1, PSCA, SLURP1***	(20)
	143,899,694–143,999,285	q24.3	99.6	20	75%	***GML***	(3)
chr20:	55,633,244–55,734,303	q13.32	101	26	70%		(2)
	55,744,225–55,754,966	q13.32	10.7	10	70%	***BMP7***	(1)
	59,550,109–59,777,020	q13.33	226.9	49	70%		(1)
	59,785,516–59,832,199	q13.33	46.7	8	70%	***CDH4***	(1)
	61,160,156–61,505,547	q13.33	345	32	70%	***NTSR1, OGFR***	(15)
	62,246,579–62,376,958	q13.33	130	24	70%	***STMN3, RTEL1, ZGAPT, SLC2A4RG, ARFRP1, TNFRSF6B***	(9)
**LOH**							
chr9:	7,641,255–9,061,636	p24.1–p23	1420.4	433	75%	***PTPRD1***	(2)
	9,415,137–17,868,117	p23–p22.2	8453	2138	75%	***PTPRD1, TYRP1, NFIB, ZDHHC21, CER1, PSIP1, BNC2, SH3GL2***	(16)
	19,032,340–20,480,069	p22.1–p21.3	1447.7	290	75%	*PLIN2, **RPS6, MLLT3***	(9)
	21,060,888–21,143,835	p21.3	82.9	25	75%		(2)
	21,370,303–21,505,928	p21.3	135.6	19	75%		(5)
	22,902,094–23,164,592	p21.3	262.5	37	75%		(0)
	25,043,325–27,458,065	p21.3–p21.2	2414.7	539	75%	***TUSC1,TEK, NCRNA00032***	(9)
	30,978,599–31,917,398	p21.1	938.8	190	70%		(0)
chr17:	0–6,858,022	p13.3–p13.1	6858	988	80%	***RPH3AL, FAM57A, GEMIN4, YWHAE, CRK, SERPINF2, SERPINF1, SMYD4, RPA1, HIC1, MNT, TM4SF5, NUP88, XAF1SMYD4, RPA1, HIC1, MNT, P2RX5, CAMKK1, ATP2A3, CYB5D2, MYBBP1A, ALOX15, PELP1, TM4SF5, PLD2, SLC25A11, RNF167, PFN1, USP6, NUP88, DHX33, XAF1, ALOX12***	(132)
	9,849,290–17,282,737	p13.1–p11.2	7433.5	1343	80%	***MYH1, MYH3, MAP2K4, MYOCD, NCOR1, ZNF624, TNFRSF13B, MPRIP, FLCN***	(57)
chr18:	28,634,894–29,240,123	q12.1	605	78	70%		(1)
	29,328,366–31,452,672	q12.1–q12.2	2124	147	70%	***MAPRE2***	(10)
	33,893,503–36,183,722	q12.2–q12.3	2290	415	75%		(1)
	36,600,581–40,107,505	q12.3	3506.9	657	75%	***PIK3C3***	(4)
	41,807,620–42,006,021	q21.1	198.4	18	75%		(3)
	61,651,678–66,668,993	q22.1–q22.2	5017	1036	70%	***CDH19, SOCS6***	(9)

*Only cancer-associated genes (in bold) or genes related to pancreas (underlined) are shown.

In adittion, large chromosomal regions displaying LOH were also detected in most PDAC tumors (80% of cases) at chromosomes 17p13.1–17p11.2 (7.5 Mb) and 17p13.3–17p13.1 (6.9 Mb) (Table 3). Noteworthy, the latter region showed complex patterns of genetic changes including LOH (10/20 cases; 50%), cnLOH (3/20 cases, 15%) and cnLOH associated with total or partial CN gains (3/20 cases, 15%). Additional LOH regions were identified on chromosome 9p (n = 8 regions that expanded from 9p21.1 to the 9p24.1 chromosome band), and chromosome 18q (n = 4 regions at 18q12.1–18q12.3 and another 2 regions in 18q21.1 and 18q22.1–18q22.2, respectively). Interestingly, LOH at chromosome 9p mainly involved regions associated with deletions of one copy of specific chromosomal areas, while LOH at chromosome 18q displayed more complex patterns of LOH in association with cnLOH.

### Correlation between the chromosomal changes detected by SNP-arrays and iFISH

Overall our results showed a high degree of correlation between the SNP-array and iFISH results with a mean R2 of 0.65±008 (range: 0.55 to 0.79) between both methods (Table 2). In addition, chromosomal abnormalities identified in those chromosomal regions evaluated by iFISH were similar to those found by SNP-array studies: gains/amplifications at 2p24 were detected in 30% of the cases by iFISH versus 20% by SNP-arrays studies (R2 = 0.70; p<0.001) as well as at 5q31 found in 25% of cases by iFISH vs. 30% by SNP-arrays studies (R2 = 0.60; p<0.005) and 8q34 (55% Vs. 50%; R2 = 0.79; p<0.001); similarly, gains/amplifications and deletions of 7q31 (20% and 20% vs. 5% and 10%, respectively; R2 = 0.62; p<0.004), 10q23 (10% and 20% vs. 5% and 10%; R2 = 0.70; p<0.001), 12p13 (20% and 45% vs. 15% and 25%; R2 = 0.76; p<0.001), 13q34 (25% and 15% vs. 55% and 10%; R2 = 0.55; p<0.012), 17q11 (15% and 10% vs. 25% and 20%; R2 = 0.59; p<0.006); similarly, deletions of chromosomes 6q21 (50% vs. 40%, respectively; R2 = 0.76; p<0.001), 6q23 (35% vs. 40%; R2 = 0.56; p<0.01), 18q21 (75% vs. 49%; R2 = 0.64; p<0.002) and 21q22 (45% vs. 40%; R2 = 0.60; p = 0.005), were detected at similar frequencies with both methods.

### Cancer-associated genes coded in chromosomal regions frequently altered in PDAC

By integrating the genomic public data (Ensembl relase 59, Human build GRCh37) with our CN and LOH results, we sought to identify regions which showed recurrent CN changes containing at least one known and well-characterized gene (Table 4). Accordingly, CN gains were frequently detected (≥75%) in those chromosomal regions coding for the *PSCA, SLURP1, NTSR1, CDH4, BAI1, TARS2, GML, OGFR* and *PTPRN2* genes, which have been described to be involved in cancer and/or pancreatic functions. Remarkably, from these genes, the PSCA, NTSR1, OGFR and *TNFRSF6B* genes have also been associated with pancreatic malignancies and the sonic hedgehog gene has been directly related to stem-cellness. In turn, the most commonly deleted gene was *MYOCD*, a cancer related gene which also displayed LOH in most of our cases (80%; Table 3); other frequently deleted cancer-associated genes included the *EYA3, NR2C1, PTAFR*, and the *DCC* cancer associated genes which have also been involved in pancreatic cancer. In turn, common regions of LOH also included two genes that have been involved in pancreatic cancer: the *RPH3AL* and *SERPINF1* genes at chromosome 17p13. Noteworthy, regions of LOH identified in chromosome 18q12 also contain genes that have been reported to be involved in pancreatic malignant tumors, e.g. the *MAPRE2* gene found to be deleted by LOH in 50% of the cases and by cnLOH in another 20% of the tumors. Other cancer-associated genes coded in those chromosomal regions displaying LOH in a relatively high proportion of cases are listed in Table 3.

**Table 4:** Genes most commonly gained and lost in PDAC: chromosomal localization and altered frequencies (n = 20).

			CN gene alteration					
Gene	Coded name	Chromosomal localization	Gains	High Copy Gain	Total Gains	Heterozygous deletion	Homozygous deletion	
Prostate stem cell antigen	*PSCA*	8q24.2	45%	35%	***80%***	0%	0%	0%
Secreted LY6/PLAUR domain containing 1	*SLURP1*	8q24.3	45%	35%	***80%***	0%	0%	0%
Neurotensin receptor 1	*NTSR1*	20q13	45%	35%	***80%***	0%	0%	0%
Cadherin 4, type 1, R-cadherin	*CDH4*	20q13.3	40%	35%	***75%***	0%	0%	0%
Brain-specific angiogenesis inhibitor 1	*BAI1*	8q24	40%	35%	***75%***	0%	0%	0%
Threonyl-tRNA synthetase 2, mitochondrial	*TARS2*	1q21.3	60%	15%	***75%***	0%	0%	0%
Glycosylphosphatidylinositol anchored molecule like protein	*GML*	8q24.3	50%	25%	***75%***	0%	0%	0%
Opioid growth factor receptor	*OGFR*	20q13.33	40%	35%	***75%***	0%	0%	0%
Protein tyrosine phosphatase, receptor type, N polypeptide 2	*PTPRN2*	7q36	35%	35%	***70%***	10%	0%	10%
Bone morphogenetic protein 7	*BMP7*	3q42	65%	5%	***70%***	0%	0%	0%
PRP3 pre-mRNA processing factor 3 homolog	*PRPF3*	1q21.1	60%	10%	***70%***	0%	0%	0%
Extracellular matrix protein 1	*ECM1*	1q21	55%	15%	***70%***	0%	0%	0%
Protein tyrosine phosphatase type IVA, member 3	*PTP4A3*	8q24.3	50%	20%	***70%***	0%	0%	0%
Stathmin-like 3	*STMN3*	3q43	35%	35%	***70%***	5%	0%	5%
Regulator of telomere elongation helicase 1	*RTEL1*	20q13.3	35%	35%	***70%***	5%	0%	5%
Zinc finger, CCCH-type with G patch domain	*ZGPAT*	20q13.3	35%	35%	***70%***	5%	0%	5%
SLC2A4 regulator	*SLC2A4RG*	20q13.33	35%	35%	***70%***	5%	0%	5%
ADP-ribosylation factor related protein 1	*ARFRP1*	20q13.3	35%	35%	***70%***	5%	0%	5%
Tumor necrosis factor receptor superfamily, member 6b, decoy	*TNFRSF6B*	20q13.3	35%	35%	***70%***	5%	0%	5%
Dipeptidyl-peptidase 6	*DPP6*	7q36.2	50%	10%	***60%***	10%	0%	10%
Sonic hedgehog	*SHH*	7q36	60%	0%	***60%***	0%	0%	0%
Laminin, alpha 5	*LAMA5*	20q13.2–q13.3	40%	20%	***60%***	0%	0%	0%
Insulin-like growth factor 1 receptor	*IGF1R*	15q26.3	30%	25%	***55%***	0%	0%	0%
Baculoviral IAP repeat-containing 7	*BIRC7*	20q13.3	15%	40%	***55%***	5%	0%	5%
Endosulfine alpha	*ENSA*	1q21.3	55%	0%	***55%***	0%	0%	0%
Epidermal growth factor receptor	*EGFR*	7p12	25%	25%	***50%***	0%	0%	0%
Myocardin	*MYOCD*	17p11.2	0%	0%	**0%**	75%	0%	***75%***
Eyes absent homolog 3	*EYA3*	1p36	0%	0%	**0%**	70%	0%	***70%***
Nuclear receptor subfamily 2, group C, member 1	*NR2C1*	12q22	0%	0%	**0%**	70%	0%	***70%***
Platelet-activating factor receptor	*PTAFR*	1p35–p34.3	0%	0%	**0%**	70%	0%	***70%***
Deleted in colorectal carcinoma	*DCC*	18q21.3	0%	0%	**0%**	70%	0%	***70%***
Myeloid/lymphoid or mixed-lineage leukemia; translocated to, 3	*MLLT3*	9p22	0%	0%	**0%**	60%	5%	***65%***
Cadherin 7, type 2	*CDH7*	18q22.1	0%	0%	**0%**	65%	0%	***65%***
Basonuclin 2	*BNC2*	9p22.2	0%	0%	**0%**	60%	0%	***60%***
Cyclin-dependent kinase inhibitor 2A	*CDKN2A*	9p21	0%	0%	**0%**	55%	5%	***60%***
Cadherin 19, type 2	*CDH19*	18q22.1	0%	0%	**0%**	60%	0%	***60%***
Replication protein A1, 70kDa	*RPA1*	17p13.3	0%	0%	**0%**	55%	0%	***55%***
TEK tyrosine kinase, endothelial	*TEK*	9p21	0%	0%	**0%**	50%	5%	***55%***
Phosphoinositide-3-kinase, class 3	*PIK3C3*	18q12.3	0%	0%	**0%**	55%	0%	***55%***

### Association between the CNV and LOH profile of PDAC tumors and other disease characteristics

Univariate analysis revealed a significant association between gains of the 10q26.13–q26.3, 11q13.1–q13.4 and 22q13.1–q13.32 chromosomal regions and grade I/II tumors (p≤0.03). Similarly, a significant association was found between cases with gains at the 10q26.13, 10q26.3, 11q13.1and 22q13.1–q13.32 chromosomal regions and smaller tumor sizes (average tumor size of 2.5 cm vs. 3.4 cm; p≤0.04), whereas Xp22.33 losses were associated with larger tumors (median tumor size of 3.5 cm vs. 2.6 cm; p = 0.03) (Table S2). No other significant associations were found between specific genetic changes and other disease features (gender, age, CA19.9 serum levels, localization of primary tumor, TNM stage, LNR, perineural invasion, vascular invasion and type of surgical resection).

**Figure 1:**
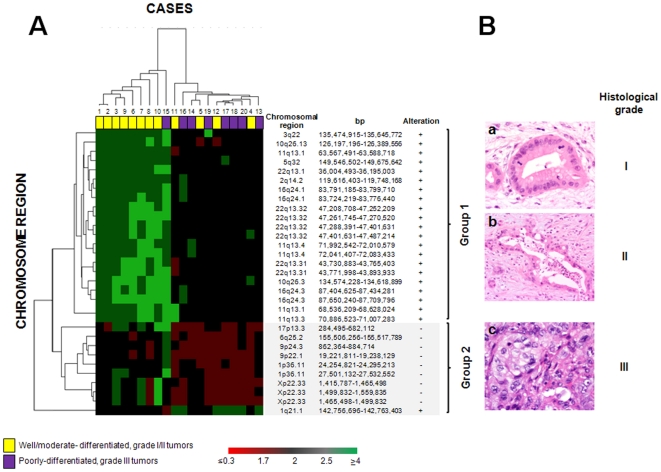
Association between tumor specific cytogenetic profiles and histopathological features. **Panel A:** Hierarchical clustering analysis of the CNV and LOH genetic profile of PDAC cases defined by the Affymetrix 500 K SNP-array (n = 20). Two well-defined groups of patients (p = 0.03) were identified: group 1 includes tumors showing gains of chromosomes 2q, 3q, 5q, 10q, 11q, 16q and 22q and a high rate of grade I/II PDAC tumors (tumor cases highlighted in yellow), while group 2 predominantly included cases with losses of chromosomes 1p, 6q, 9p, 17p and Xp, and gains of chromosome 1q, in association with a higher frequency of poorly-differentiated/grade III carcinomas (tumor cases highlighted in purple). CNV obtained for each chromosomal region are represented in a color code: red corresponds to chromosomal losses (CN≤1.7), green to chromosomal gains (CN≥2.5) and black to a normal CN value of 2. The color intensity represents the magnitude of the change, down to CN values <0.3 (homozygous deletions) and up to CN values ≥4 (high gains/amplification). Known cancer-associated genes coded in these chromosomal regions and found to be altered include the *INPP5A* (at 10q26.3), *CDX1, CAMK2A* (at 5q32), *MB* and *APOL6* (both at 22q13.1) genes gained among group 1 cases and the *SFRS13A* (1p36.11), *VPS53, FAM57A, GEMIN4, ELP2P* and *GLOD4* (all of them at 17p13.3) and the *CSF2RA* and the *IL3RA* (both at Xp22.33) genes deleted in group 2 cases. **Panel B:** Illustrating histopathological pictures corresponding to group 1 -histologic grades I and II PDACs characterized by typically well-formed glands and less well-defined glands with an incomplete glandular lumina, respectively (images **a** and **b**)-, and group 2 - grade III PDAC showing non-structured glands and solid sheets of neoplastic cells (c)- PDAC cases. Original magnification: (**a**) x200; (**b**) x100; (**c**) x400.

Hierarchical clustering analysis of the 20 PDAC tumors studied according to their CNV and LOH profiles showed the existence of two well-defined groups of tumors (Figure 1). All 9 PDAC tumors from group 1 showed gains of the 2q14.2, 3q22.1, 5q32, 10q26.13, 10q26.3, 11q13.1, 11q13.3, 11q13.4, 16q24.1, 16q24.3, 22q13.1, 22q13.31 and 22q13.32 chromosomal regions, while group 2 consisted of 11 cases who shared gains of the 1q21.1 chromosomal region together with a relatively high frequency of losses (≥64% of cases) of the 1p36.11, 6q25.2, 9p22.1, 9p24.3, 17p13.3 and for Xp22.33 chromosomal regions. Interestingly, the former group also showed a high rate of well/moderate-differentiated grade I/II PDAC tumors (8/9 cases, 89%; p = 0.03), while group 2 patients mainly corresponded to poorly-differentiated, grade III carcinomas (8/11 cases, 73%; p = 0.03) (Figure 1). Twelve cancer-associated genes mapping in six of these regions were identified to be altered: the *INPP5A* (at 10q26.3), *CDX1*, *CAMK2A* (at 5q32), *MB* and *APOL6* (both at 22q13.1) genes among the former (group 1) cases, and the *SFRS13A* (1p36.11), *VPS53*, *FAM57A*, *GEMIN4*, *ELP2P* and *GLOD4* (all of them at 17p13.3) and the CSF2RA and the IL3RA (both at Xp22.33) genes, all deleted among group 2 cases (Figure 1) .

## Discussion

PDAC are heterogeneous tumors that frequently display complex genetic profiles as confirmed in the present study where multiple CNV and LOH regions were identified in every case analyzed. Overall, our findings indicate that the genetic profile of primary PDAC is defined by imbalanced losses of chromosomes 1p, 9p, 12q, 17p and 18q together with gains of the 1q, 7q, 8q and 20q chromosomal regions. These results confirm previous analyses using chromosome banding techniques[13], [14], CGH[15], aCGH[1], [2], [4], [16], low-resolution 100K SNP-arrays[8] and gene sequencing combined or not with SNP-array technology [9], [17]. Despite a high correlation was also found between the SNP-array results and iFISH analyses performed on the same series of primary tumors samples - as regards the most commonly deleted (e.g. 17p, 18q, 9p and 8p) and gained (e.g. 1q, 15q and 8q) chromosomal regions[11]-, a higher frequency of deletions at chromosomes 1p and 17q, and gains at chromosomes 7q and 20q were found by SNP- arrays vs. iFISH technique (around 70–75% vs. 5–25%, respectively). Such discrepancies could be explained, at least in part, by the increased sensitivity of the SNP-array vs. iFISH studies in the identification of small interstitial changes[18].

A more detailed analysis of the most frequently altered chromosomal regions shows that they contain multiple cancer-associated genes, including several genes which have been specifically related to PDAC. Among others, these latter genes consisted of gained genes such as the *PSCA* gene, a plausible PDAC tumor marker associated with pancreatic cancer progression[19]–[22], the *TNFRSF6B* gene (a member of the tumor necrosis factor receptor family) which is amplificated in many tumors [23]–[26] and whose overexpression blocks growth inhibition signals in PDAC[27], and the *NTSR1* and *OGFR* genes, involved in cancer progression[28]–[30], modulation of angiogenesis[31] and regulation of cell proliferation[32]. In turn, frequently deleted genes of interest were the *RPH3AL* gene, a potential tumor suppressor gene related with insulin exocytosis[33], the *SERPINF1* gene which has been detected to be involved in many epithelium derived tumors[34], [35], and the *MAPRE2* gene, previously found to be lost in leukemic cells[36], pancreatic cancer[37] and esophageal squamous cell carcinoma[38]; interestingly, deletion of other cancer associated genes which have not been previously associated to pancreatic malignances (*MYOCD* [39], [40], *NR2C1* [41] and *PTAFR* [42]) were found at higher frequencies than other (e.g. *CDKN2A*, *TP53* or *SMAD4* [43], [44]) genes shown to be recurrently altered/deleted in PDAC. These results underline the potential role of several previously unexplored tumor suppressor genes in the pathogenesis of PDAC. In turn, genes which have been previously found to be amplified in PDAC patients by SNP-arrays[8], such as the *SACP2* gene, were also altered in our series but at a lower frequency (e.g. 60% vs. 40% of cases, respectively). Such variability could be partially related to the lower number of patients analyzed and the effect of studying paired tumoral/normal DNA samples in the resolution of the SNP-array for detection of CN alterations.

Most interestingly, is the observation that based on the overall genetic profile of PDAC tumors detected by SNP-arrays two well defined groups of PDAC tumors emerge which are differentially characterized by gains of the 2q14.2, 3q22.1, 5q32, 10q26.13, 10q26.3, 11q13.1, 11q13.3, 11q13.4, 16q24.1, 16q24.3, 22q13.1, 22q13.31 and 22q13.32 chromosomal regions (group 1) and by gains at 1q21.1 with coexisting losses of the 1p36.11, 6q25.2, 9p22.1, 9p24.3, 17p13.3 and Xp22.33 chromosomal regions (group 2), respectively. From the clinical and histopathologicall point of view, while group 1 PDAC mostly corresponded to smaller well/moderately differentiated grade I/II cases, group 2 mainly consisted of larger and poorly-differentiated PDAC. Among the few well/moderately differentiated carcinomas included in this latter group, 2/3 cases showed intermediate cytogenetic features with coexistence of gains of chromosomes 1q21.1 together with gains of chromosomes 10q, 22q and 11q. Whether these two distinct cytogenetic profiles reflect different cytogenetic pathways vs. sequential stages of development of PDAC, remains to be determined. However, the identification of rather different and non-overlapping chromosomal changes in both groups of tumors would support they could more likely reflect two genetically different diseases. Further studies in larger series of patients are warranted to elucidate this question and determine the specific role of those cancer associated genes (*INPP5A, CDX1, MB, CAMK2A, APOL6* vs. *VPS53, FAM57A, GEMIN4, SFRS13, ELP2P, GLOD4, CSF2RA* and *IL3RA*), differentially altered in both groups of tumors. In this regard, it should be noted that from those genes, two or more are involved in common intracellular pathways such the cytokine-cytokine receptor interactions involving Jak-STAT signaling (*IL3RA* and *CSF2RA* genes) or RNA processing pathways (*GEMIN4* and *SFRS13A* genes) [45], [46]. Further analyses of gene expression profiles may contribute to determine their relevance in the pathogenesis of PDAC.

In summary, in the present study we confirm the cytogenetic complexity and heterogenity of PDAC and provide evidence for the association between tumor cytogenetics and its histopathological features. In addition, we also show that the most frequently altered regions identified harbor multiple cancer-associated genes that deserve further investigation to determine their relevance in the pathogenesis of PDAC.

## Supporting Information

Table S1 Frequently detected regions (≤65%) of gain, loss and LOH in PDAC tumors (n = 20) genotyped on the Affymetrix 500 K SNP array platform.
(DOC)

Table S2 Association between specific CN alterations found in PDAC tumors (n = 20) and both tumor size and histopathology.
(DOC)
