# Supplementary material for: Association between Genetic Subgroups of Pancreatic Ductal Adenocarcinoma Defined by High Density 500 K SNP-Arrays and Tumor Histopathology
Source: PLoS One. 2011 Jul 21;6(7):e22315. doi: 10.1371/journal.pone.0022315 (PMC3141022; doi:10.1371/journal.pone.0022315)
Supplement: Table S1 — Frequently detected regions (≤65%) of gain, loss and LOH in PDAC tumors (n = 20) genotyped on the Affymetrix 500 K SNP array platform. (DOC) [file pone.0022315.s001.doc]

Table S1: Frequently detected regions (≤65%) of gain, loss and LOH in PDAC tumors (n=20) genotyped on the Affymetrix 500K SNP array platform.

| **Chromosomal (Chr) region (bp)** | **Chromosome band** | **Length (Kb)** | **% of altered cases** |
| --- | --- | --- | --- |
| **CN Losses** |  |  |  |
| chr1: 28,245,012-28,862,857 | p35.3 | 617.8 | 50 |
| chr1: 31,292,882-31,296,438 | p35.2 | 3.6 | 50 |
| chr6: 5,347,933-5,473,881 | p25.1 | 125.9 | 50 |
| chr6: 114,025,343-114,082,601 | q21 | 57.3 | 60 |
| chr8: 7,222,992-7,278,542 | p23.1 | 55.6 | 55 |
| chr8: 33,300,800-33,304,753 | p12 | 4 | 55 |
| chr8: 33,330,093-33,454,016 | p12 | 123.9 | 55 |
| chr9: 74,391,174-74,493,473 | q21.13 | 102.3 | 50 |
| chr10: 73,920,768-74,004,764 | q22.1 | 84 | 50 |
| chr10: 75,985,568-76,043,117 | q22.2 | 57.6 | 50 |
| chr10: 76,068,446-76,108,090 | q22.2 | 39.6 | 50 |
| chr11: 50,301,567-50,339,013 | p11.12 | 37.5 | 50 |
| chr12: 1,112,075-1,171,662 | p13.33 | 59.6 | 50 |
| chr17: 54,264,925-54,463,325 | q23.2 | 198.4 | 55 |
| chr17: 56,183,184-56,276,756 | q23.2 | 93.6 | 55 |
| chrX: 1,465,498-1,499,832 | p22.33 | 34.3 | 50 |
| **CN Gains** |  |  |  |
| chr1: 742,429-789,326 | p36.33 | 46.9 | 65 |
| chr1: 2,017,761-2,100,120 | p36.33 | 82.4 | 50 |
| chr1: 2,310,562-2,347,670 | p36.32 | 37.1 | 50 |
| chr1: 2,408,485-2,543,618 | p36.32 | 135.1 | 50 |
| chr1: 2,703,187-3,502,376 | p36.32 | 799.2 | 50 |
| chr1: 4,527,839-4,639,285 | p36.32 | 111.5 | 50 |
| chr1: 5,956,787-6,099,789 | p36.31 | 143 | 50 |
| chr1: 41,622,769-41,781,591 | p34.2 | 158.8 | 50 |
| chr1: 41,866,299-41,867,246 | p34.2 | 0.95 | 50 |
| chr1: 53,670,105-53,737,764 | p32.3 | 67.7 | 50 |
| chr1: 146,183,314-146,292,286 | q21.1 | 109 | 65 |
| chr1: 147,188,028-147,521,567 | q21.2 | 333.5 | 65 |
| chr1: 150,306,335-150,496,340 | q21.3 | 190 | 65 |
| chr1: 159,730,122-159,745,375 | q23.3 | 15.3 | 65 |
| chr2: 186,973-200,076 | p25.3 | 13.1 | 55 |
| chr2: 214,086-357,736 | p25.3 | 143.7 | 55 |
| chr2: 406,815-671,521 | p25.3 | 264.7 | 55 |
| chr2: 695,636-716,030 | p25.3 | 20.4 | 60 |
| chr2: 994,430-1,027,581 | p25.3 | 33.2 | 55 |
| chr2: 1,038,118-1,213,866 | p25.3 | 175.8 | 55 |
| chr2: 1,257,125-1,484,084 | p25.3 | 227 | 55 |
| chr2: 1,536,934-1,972,031 | p25.3 | 435.1 | 55 |
| chr2: 2,583,817-2,657,145 | p25.3 | 73.3 | 55 |
| chr2: 2,675,835-2,895,962 | p25.3 | 220.1 | 55 |
| chr2: 3,206,115-3,259,849 | p25.3 | 53.7 | 55 |
| chr2: 3,704,731-3,727,421 | p25.3 | 22.7 | 55 |
| chr2: 3,737,705-3,805,968 | p25.3 - p25.2 | 68.3 | 55 |
| chr2: 3,813,933-3,817,006 | p25.2 | 3.1 | 55 |
| chr2: 20,669,934-20,704,649 | p24.1 | 34.7 | 55 |
| chr2: 26,532,000-26,677,750 | p23.3 | 145.8 | 55 |
| chr2: 71,647,874-71,784,206 | p13.2 | 136.3 | 55 |
| chr2: 71,815,229-71,860,972 | p13.2 | 45.7 | 55 |
| chr2: 73,359,349-73,359,413 | p13.2 | 0.064 | 55 |
| chr2: 94,901,299-94,914,685 | q11.1 | 13.4 | 50 |
| chr2: 95,946,534-96,200,239 | q11.2 | 253.7 | 50 |
| chr2: 98,562,915-98,742,232 | q11.2 | 179.3 | 50 |
| chr2: 109,395,730-109,452,923 | q13 | 57.2 | 50 |
| chr2: 119,616,403-119,748,168 | q14.2 | 131.8 | 50 |
| chr2: 120,852,125-121,525,367 | q14.2 | 673.2 | 50 |
| chr2: 156,855,395-156,972,295 | q24.1 | 117 | 50 |
| chr2: 218,370,272-218,376,045 | q35 | 5.8 | 50 |
| chr2: 218,388,836-218,405,194 | q35 | 16.4 | 50 |
| chr2: 218,415,998-218,552,243 | q35 | 136.3 | 50 |
| chr2: 218,572,564-218,604,736 | q35 | 32.2 | 50 |
| chr2: 237,019,497-237,025,233 | q37.2 | 5.7 | 55 |
| chr2: 238,189,155-238,312,207 | q37.3 | 123.1 | 50 |
| chr2: 238,928,000-238,930,959 | q37.3 | 3 | 50 |
| chr2: 238,953,094-239,180,160 | q37.3 | 227.1 | 50 |
| chr2: 239,506,202-239,846,809 | q37.3 | 340.6 | 50 |
| chr2: 239,983,692-240,667,487 | q37.3 | 683.8 | 50 |
| chr2: 240,691,407-240,772,862 | q37.3 | 81.5 | 50 |
| chr2: 240,775,988-240,789,869 | q37.3 | 13.9 | 50 |
| chr2: 240,799,649-240,975,487 | q37.3 | 175.8 | 50 |
| chr2: 240,993,845-241,030,756 | q37.3 | 36.9 | 50 |
| chr2: 241,091,399-241,113,588 | q37.3 | 22.2 | 50 |
| chr2: 241,276,509-241,545,091 | q37.3 | 268.6 | 50 |
| chr2: 242,567,344-242,663,303 | q37.3 | 96 | 55 |
| chr3: 10,820,313-10,891,332 | p25.3 | 71.0 | 50 |
| chr3: 10,897,741-11,061,367 | p25.3 | 163.6 | 50 |
| chr3: 13,803,222-13,882,158 | p25.1 | 78.9 | 50 |
| chr3: 127,080,119-127,185,609 | q21.2 | 105.5 | 50 |
| chr3: 127,203,704-127,337,850 | q21.2 | 134.2 | 50 |
| chr3: 127,350,140-127,936,525 | q21.2 | 586.4 | 50 |
| chr3: 128,125,240-128,195,448 | q21.2 | 70.2 | 50 |
| chr3: 128,226,897-128,240,195 | q21.2 | 13.3 | 50 |
| chr3: 128,254,303-128,772,830 | q21.2 - q21.3 | 518.5 | 50 |
| chr3: 129,469,163-129,673,631 | q21.3 | 204.5 | 50 |
| chr3: 130,715,044-130,822,332 | q21.3 | 107.3 | 50 |
| chr3: 135,402,599-135,439,955 | q22.1 | 37.4 | 50 |
| chr3: 135,474,915-135,645,772 | q22.1 | 170.9 | 50 |
| chr3: 142,374,603-142,449,139 | q23 | 74.5 | 50 |
| chr3: 144,324,720-144,339,430 | q23 | 14.7 | 50 |
| chr3: 144,345,081-144,370,150 | q23 | 25.1 | 50 |
| chr3: 147,370,449-147,498,076 | q24 | 127.6 | 50 |
| chr3: 180,292,559-180,367,031 | q26.32 | 74.5 | 50 |
| chr4: 3,379,157-3,731,921 | p16.3 | 352.8 | 50 |
| chr4: 7,336,346-7,432,332 | p16.1 | 96 | 50 |
| chr4: 7,510,244-7,666,095 | p16.1 | 155.9 | 50 |
| chr4: 81,368,193-81,471,743 | q21.21 | 103.6 | 50 |
| chr4: 148,836,619-148,836,997 | q31.23 | 0.38 | 50 |
| chr4: 190,816,289-190,878,050 | q35.2 | 61.8 | 50 |
| chr4: 190,915,100-190,945,867 | q35.2 | 30.8 | 50 |
| chr5: 734,302-752,190 | p15.33 | 17.9 | 55 |
| chr5: 771,021-797,863 | p15.33 | 26.8 | 55 |
| chr5: 872,671-949,726 | p15.33 | 77.1 | 55 |
| chr5: 971,812-1,630,411 | p15.33 | 658.6 | 55 |
| chr5: 1,661,591-1,997,487 | p15.33 | 335.9 | 55 |
| chr5: 2,012,810-2,249,941 | p15.33 | 237.1 | 55 |
| chr5: 2,267,721-2,271,094 | p15.33 | 3.4 | 55 |
| chr5: 2,544,676-2,904,756 | p15.33 | 360.1 | 55 |
| chr5: 2,919,892-2,931,144 | p15.33 | 11.3 | 55 |
| chr5: 3,382,278-3,767,522 | p15.33 | 385.2 | 55 |
| chr5: 3,787,365-3,796,669 | p15.33 | 9.3 | 55 |
| chr5: 6,443,651-6,558,467 | p15.31 | 114.8 | 55 |
| chr5: 6,752,224-6,839,308 | p15.31 | 87.1 | 55 |
| chr5: 10,520,257-10,801,683 | p15.2 | 281.4 | 55 |
| chr5: 14,544,544-14,588,828 | p15.2 | 44.3 | 55 |
| chr5: 16,243,953-16,317,285 | p15.1 | 73.3 | 55 |
| chr5: 134,430,895-134,489,380 | q31.1 | 58.5 | 50 |
| chr5: 149,546,502-149,675,642 | q32 | 129.1 | 50 |
| chr5: 175,130,801-175,279,396 | q35.2 | 148.6 | 55 |
| chr5: 175,371,041-175,525,010 | q35.2 | 154 | 50 |
| chr5: 176,780,625-176,837,482 | q35.3 | 56.9 | 50 |
| chr5: 178,532,223-178,536,983 | q35.3 | 4.8 | 50 |
| chr5: 178,552,922-178,784,000 | q35.3 | 231.1 | 50 |
| chr5: 178,816,693-178,820,503 | q35.3 | 3.8 | 50 |
| chr6: 33,148,813-33,285,988 | p21.32 | 137.2 | 50 |
| chr6: 168,284,746-168,580,479 | q27 | 295.7 | 50 |
| chr6: 169,900,793-170,359,831 | q27 | 459.1 | 50 |
| chr7: 1,078,159-1,355,201 | p22.3 | 277 | 55 |
| chr7: 1,698,392-1,815,653 | p22.3 | 117.3 | 55 |
| chr7: 1,878,748-1,903,205 | p22.3 | 24.5 | 55 |
| chr7: 45,518,386-45,521,511 | p13 | 3.1 | 55 |
| chr7: 45,531,364-45,613,377 | p13 | 82. | 55 |
| chr7: 45,645,546-45,761,480 | p13 | 115.9 | 55 |
| chr7: 50,320,813-50,335,957 | p12.2 | 15.1 | 55 |
| chr7: 127,482,050-127,498,937 | q32.1 | 16.9 | 50 |
| chr7: 127,518,854-127,587,286 | q32.1 | 68.4 | 50 |
| chr7: 127,621,231-127,660,228 | q32.1 | 39 | 50 |
| chr7: 142,176,424-142,390,212 | q34 | 213.8 | 50 |
| chr7: 142,701,483-142,779,432 | q34 | 77.9 | 50 |
| chr7: 142,803,429-142,927,312 | q34 | 123.9 | 50 |
| chr7: 154,166,444-154,183,276 | q36.2 | 16.8 | 50 |
| chr7: 154,197,342-154,368,113 | q36.2 | 170.8 | 50 |
| chr7: 154,876,017-155,043,605 | q36.3 | 167.6 | 50 |
| chr7: 155,324,299-155,766,005 | q36.3 | 441.7 | 50 |
| chr7: 156,946,444-156,953,430 | q36.3 | 7 | 50 |
| chr7: 156,970,589-157,987,469 | q36.3 | 1016.9 | 50 |
| chr7: 158,520,162-158,582,043 | q36.3 | 61.9 | 50 |
| chr7: 158,601,057-158,606,931 | q36.3 | 5.9 | 50 |
| chr8: 1,037,318-1,059,260 | p23.3 | 21.9 | 50 |
| chr8: 1,091,797-1,095,978 | p23.3 | 4.2 | 50 |
| chr8: 1,278,622-1,281,480 | p23.3 | 2.9 | 50 |
| chr8: 1,292,237-1,327,236 | p23.3 | 35 | 50 |
| chr8: 41,649,639-41,674,630 | p11.21 | 25 | 50 |
| chr8: 41,682,842-41,687,028 | p11.21 | 4.2 | 50 |
| chr8: 140,737,659-140,810,733 | q24.3 | 73.1 | 65 |
| chr8: 140,825,354-140,826,636 | q24.3 | 1.3 | 65 |
| chr8: 142,069,369-142,086,091 | q24.3 | 16.7 | 65 |
| chr8: 142,107,781-142,143,028 | q24.3 | 35.3 | 65 |
| chr8: 142,153,403-142,175,410 | q24.3 | 22 | 65 |
| chr8: 142,194,487-142,257,221 | q24.3 | 62.7 | 65 |
| chr8: 142,266,863-142,343,836 | q24.3 | 77 | 65 |
| chr8: 142,371,206-143,999,285 | q24.3 | 1628.1 | 65 |
| chr8: 144,615,338-144,909,318 | q24.3 | 294 | 65 |
| chr10: 41,956,473-42,002,139 | q11.21 | 45.7 | 65 |
| chr10: 42,673,001-43,015,055 | q11.21 | 342.1 | 50 |
| chr10: 43,054,312-43,152,050 | q11.21 | 97.7 | 50 |
| chr10: 43,169,364-43,181,930 | q11.21 | 12.6 | 50 |
| chr10: 131,448,641-131,563,111 | q26.3 | 114.5 | 50 |
| chr10: 132,960,674-133,270,631 | q26.3 | 310 | 50 |
| chr10: 133,285,378-133,459,961 | q26.3 | 174.6 | 50 |
| chr10: 133,839,108-134,076,623 | q26.3 | 237.5 | 55 |
| chr10: 134,316,153-134,354,683 | q26.3 | 38.5 | 50 |
| chr10: 134,388,960-134,618,899 | q26.3 | 229.9 | 50 |
| chr11: 2,549,107-2,656,737 | p15.5 | 107.6 | 50 |
| chr11: 2,667,398-2,717,317 | p15.5 | 49.9 | 50 |
| chr11: 2,729,947-2,865,232 | p15.5 - p15.4 | 135.3 | 50 |
| chr11: 17,445,237-17,735,082 | p15.1 | 289.9 | 50 |
| chr11: 49,816,620-49,909,938 | p11.12 | 93.3 | 60 |
| chr11: 68,536,209-68,857,006 | q13.3 | 320.8 | 50 |
| chr11: 69,985,448-70,201,641 | q13.3 - q13.4 | 216.2 | 50 |
| chr11: 70,886,523-71,007,283 | q13.4 | 120.7 | 50 |
| chr11: 71,992,542-72,010,579 | q13.4 | 18. | 50 |
| chr11: 72,041,407-72,083,433 | q13.4 | 42.0 | 50 |
| chr11: 75,474,467-75,600,560 | q13.5 | 126.1 | 50 |
| chr11: 133,698,952-133,752,397 | q25 | 53.5 | 50 |
| chr12: 1,673,782-1,726,085 | p13.33 | 52.3 | 50 |
| chr12: 1,748,415-1,785,820 | p13.33 | 37.4 | 50 |
| chr12: 6,864,604-6,955,432 | p13.31 | 90.8 | 50 |
| chr12: 6,982,497-6,991,097 | p13.31 | 8.6 | 50 |
| chr12: 91,818,116-91,830,164 | q22 | 12.0 | 60 |
| chr12: 95,415,850-95,567,680 | q23.1 | 151.8 | 60 |
| chr13: 19,576,513-19,708,737 | q12.11 | 132.2 | 50 |
| chr13: 109,629,535-109,936,256 | q34 | 306.7 | 50 |
| chr13: 110,097,814-110,106,226 | q34 | 8.4 | 50 |
| chr13: 110,176,653-110,946,274 | q34 | 769.6 | 50 |
| chr13: 110,993,446-111,141,350 | q34 | 147.9 | 50 |
| chr13: 111,553,756-111,684,549 | q34 | 130.8 | 50 |
| chr13: 111,707,578-111,733,403 | q34 | 25.8 | 50 |
| chr13: 111,767,403-111,970,835 | q34 | 203.4 | 55 |
| chr13: 112,025,929-112,175,472 | q34 | 149.5 | 50 |
| chr13: 112,193,014-112,388,718 | q34 | 195.7 | 50 |
| chr13: 112,557,920-112,630,442 | q34 | 72.5 | 50 |
| chr13: 112,649,062-112,805,998 | q34 | 156.9 | 50 |
| chr13: 112,936,202-113,160,288 | q34 | 224.1 | 50 |
| chr13: 113,189,296-113,366,240 | q34 | 176.9 | 50 |
| chr13: 113,479,759-113,839,278 | q34 | 359.5 | 50 |
| chr13: 113,908,069-113,996,575 | q34 | 88.5 | 50 |
| chr14: 100,069,036-100,321,061 | q32.2 | 252 | 50 |
| chr14: 103,698,082-104,161,600 | q32.33 | 463.5 | 50 |
| chr14: 105,399,872-105,553,453 | q32.33 | 153.6 | 50 |
| chr15: 18,427,103-18,451,755 | q11.2 | 24.6 | 55 |
| chr15: 75,753,223-75,943,808 | q24.3 | 190.6 | 50 |
| chr15: 99,118,340-99,150,496 | q26.3 | 32.2 | 50 |
| chr15: 99,180,792-99,379,419 | q26.3 | 198.6 | 50 |
| chr15: 99,389,091-99,497,972 | q26.3 | 108.9 | 50 |
| chr15: 99,515,982-99,593,641 | q26.3 | 77.7 | 50 |
| chr15: 99,611,174-99,933,168 | q26.3 | 322 | 50 |
| chr16: 1,416,466-1,444,150 | p13.3 | 27.7 | 50 |
| chr16: 2,933,611-3,005,925 | p13.3 | 72.3 | 50 |
| chr16: 3,041,640-3,105,052 | p13.3 | 63.4 | 50 |
| chr16: 3,127,734-3,193,162 | p13.3 | 65.4 | 50 |
| chr16: 48,144,228-48,267,591 | q12.1 | 123.4 | 55 |
| chr16: 49,113,279-49,121,148 | q12.1 | 7.9 | 55 |
| chr16: 49,132,748-49,323,628 | q12.1 | 190.9 | 55 |
| chr16: 83,622,586-83,724,219 | q24.1 | 101.6 | 55 |
| chr16: 87,434,281-87,465,349 | q24.3 | 31.1 | 55 |
| chr16: 87,480,405-87,591,520 | q24.3 | 111.1 | 55 |
| chr18: 5,461,765-5,612,011 | p11.31 | 150.3 | 50 |
| chr20: 55,474,916-55,503,911 | q13.32 | 29 | 65 |
| chr20: 55,522,822-55,781,996 | q13.32 | 259.2 | 65 |
| chr20: 59,516,281-59,884,538 | q13.33 | 368.3 | 65 |
| chr20: 61,160,156-61,541,410 | q13.33 | 381.3 | 65 |
| chr20: 62,202,436-62,376,958 | q13.33 | 174.5 | 65 |
| chr21: 14,032,696-14,136,067 | q11.2 | 103.4 | 55 |
| chr21: 42,560,211-42,663,562 | q22.3 | 103.4 | 50 |
| chr21: 42,677,482-42,771,264 | q22.3 | 93.8 | 50 |
| chr21: 43,513,756-43,615,770 | q22.3 | 102 | 50 |
| chr21: 43,635,184-43,661,787 | q22.3 | 26.6 | 50 |
| chr21: 44,703,694-44,722,797 | q22.3 | 19.1 | 50 |
| chr21: 44,752,483-44,934,972 | q22.3 | 182.5 | 50 |
| chr21: 45,592,958-45,789,927 | q22.3 | 197 | 50 |
| chr21: 46,109,320-46,375,182 | q22.3 | 265.9 | 50 |
| chr22: 18,579,579-18,703,211 | q11.21 | 123.6 | 60 |
| chrX: 18,654-20,336 | p22.33 | 1.7 | 50 |
| chrX: 48,907,626-49,060,243 | p11.23 | 152.6 | 55 |
| chrX: 67,808,504-68,086,141 | q13.1 | 277.6 | 50 |
| chrX: 82,683,103-82,778,245 | q21.1 | 95.1 | 50 |
| chrX: 134,159,698-134,303,928 | q26.3 | 144.2 | 55 |
| chrX: 152,352,913-152,587,060 | q28 | 234.2 | 50 |
| chrX: 153,531,195-153,597,424 | q28 | 66.2 | 55 |
|  |  |  |  |
| **LOH** |  |  |  |
| chr1: 22,186,372-30,082,397 | p36.12 - p35.2 | 7896025 | 50 |
| chr3: 50,261,396-52,039,238 | p21.31 - p21.1 | 1777842 | 50 |
| chr4: 143,182,703-143,191,465 | q31.21 | 8762 | 50 |
| chr4: 143,568,791-144,582,575 | q31.21 | 1013784 | 50 |
| chr6: 671,977-7,660,631 | p25.3 - p24.3 | 6988654 | 50 |
| chr6: 62,030,184-63,239,636 | q11.1 | 1209452 | 50 |
| chr6: 63,515,660-64,620,092 | q12 | 1104432 | 50 |
| chr6: 66,553,130-66,709,579 | q12 | 156449 | 50 |
| chr6: 67,427,185-83,004,460 | q12 - q14.1 | 15577275 | 50 |
| chr6: 116,144,440-118,647,304 | q22.1 - q22.31 | 2502864 | 50 |
| chr6: 130,006,072-130,024,483 | q22.33 | 18411 | 50 |
| chr6: 134,261,945-135,638,281 | q23.2 - q23.3 | 1376336 | 50 |
| chr6: 136,277,708-149,465,362 | q23.3 - q25.1 | 13187654 | 50 |
| chr6: 149,712,981-150,196,896 | q25.1 | 483915 | 50 |
| chr6: 150,594,889-151,753,791 | q25.1 | 1158902 | 50 |
| chr8: 0-29,308,984 | p23.3 - p21.1 | 29308984 | 50 |
| chr8: 30,236,970-30,896,659 | p12 | 659689 | 50 |
| chr8: 31,052,631-35,011,700 | p12 | 3959069 | 50 |
| chr8: 35,542,647-40,374,707 | p12 - p11.21 | 4832060 | 50 |
| chr8: 50,453,619-51,102,658 | q11.22 | 649039 | 50 |
| chr8: 51,147,043-51,651,339 | q11.22 | 504296 | 50 |
| chr9: 0-35,121,046 | p24.3 - p13.3 | 35121046 | 50 |
| chr9: 35,176,767-38,761,831 | p13.3 - p13.1 | 3585064 | 50 |
| chr9: 67,813,967-69,117,359 | q12 | 1303392 | 60 |
| chr9: 73,079,819-75,969,016 | q21.12 - q21.13 | 2889197 | 50 |
| chr9: 81,294,514-81,907,567 | q21.31 | 613053 | 50 |
| chr10: 20,654,212-21,362,004 | p12.31 | 707792 | 50 |
| chr10: 22,608,463-23,267,669 | p12.31 - p12.2 | 659206 | 50 |
| chr10: 74,213,061-75,326,562 | q22.1 - q22.2 | 1113501 | 50 |
| chr10: 101,271,104-101,314,390 | q24.2 | 43286 | 50 |
| chr11: 36,580,175-40,300,342 | p12 | 3720167 | 50 |
| chr12: 0-6,497,378 | p13.33 - p13.31 | 6497378 | 50 |
| chr12: 6,983,416-24,075,781 | p13.31 - p12.1 | 17092365 | 50 |
| chr12: 24,896,965-28,324,350 | p12.1 - p11.22 | 3427385 | 50 |
| chr12: 28,628,279-29,012,019 | p11.22 | 383740 | 50 |
| chr12: 29,527,159-29,569,650 | p11.22 | 42491 | 50 |
| chr12: 30,074,169-30,247,492 | p11.22 | 173323 | 50 |
| chr12: 31,881,221-31,976,921 | p11.21 | 95700 | 50 |
| chr12: 71,534,606-132,349,534 | q21.1 - q24.33 | 60814928 | 50 |
| chr16: 33,847,701-34,996,986 | p11.2 - p11.1 | 1149285 | 50 |
| chr17: 47,251,746-48,948,508 | q21.33 - q22 | 1696762 | 50 |
| chr17: 49,023,077-49,046,279 | q22 | 23202 | 50 |
| chr17: 49,548,805-51,007,003 | q22 | 1458198 | 50 |
| chr17: 67,197,337-69,603,020 | q24.3 - q25.1 | 2405683 | 50 |
| chr18: 16,100,000-16,863,806 | q11.1 | 763806 | 50 |
| chr18: 20,358,489-76,117,153 | q11.2 - q23 | 55758664 | 50 |
