# Supplementary material for: Association between Genetic Subgroups of Pancreatic Ductal Adenocarcinoma Defined by High Density 500 K SNP-Arrays and Tumor Histopathology
Source: PLoS One. 2011 Jul 21;6(7):e22315. doi: 10.1371/journal.pone.0022315 (PMC3141022; doi:10.1371/journal.pone.0022315)
Supplement: Table S2 — Association between specific CN alterations found in PDAC tumors (n = 20) and both tumor size and histopathology. (DOC) [file pone.0022315.s002.doc]

Table S2: Association between specific CN alterations found in PDAC tumors (n=20) and both tumor size and histopathology.

|  |  |  |  |  |  | **Tumor Size** | | | **Histological grade** | | |
| --- | --- | --- | --- | --- | --- | --- | --- | --- | --- | --- | --- |
| **Chromosome band** | **Extent of the chromosomal region (bp)** | **CN alteration** | **Length (Kb)** | **Genes*** | **% of cases with alteration** | **Tumor size of altered cases^§^** | **Tumor size of non-altered cases^§^** | **p-Value** | **% of grade I/II cases** | **% of grade III cases** | **p-Value** |
| 10q26.13 | 126,197,196-126,389,556 | Gain | 192.4 | *LHPP, FAM53B* | 45 | 2.55±0.53 | 3.43±1.0 | **0.04** | 67 | 12.5 | **0.03** |
| 10q26.3 | 134,574,228-134,618,899 | Gain | 44.7 | *NKX6-2,* ***INPP5A*** | 50 | 2.52±0.51 | 3.57±1.0 | **0.007** | 75 | 12.5 | **0.02** |
| 11q13.1 | 63,567,491-63,588,718 | Gain | 21.2 | *C11orf84* | 45 | 2.55±0.53 | 3.43±1.0 | **0.04** | 67 | 12.5 | **0.03** |
| 11q13.3 | 68,536,209-68,628,024 | Gain | 91.8 | *CPT1A* | 50 | 2.65±0.58 | 3.42±1.1 | 0.07 | 75 | 12.5 | **0.02** |
| 11q13.4 | 70,886,523-71,007,283 | Gain | 120.8 | *SHANK* | 50 | 2.65±0.58 | 3.42±1.1 | 0.07 | 75 | 12.5 | **0.02** |
| 22q13.1 | 36,004,493-36,195,003 | Gain | 190.5 | ***MB****,* ***APOL6****, APOL5, RBFOX2* | 45 | 2.55±0.53 | 3.43±1.0 | **0.04** | 67 | 12.5 | **0.03** |
| 22q13.31 | 43,730,883-43,765,403 | Gain | 34.5 | *SCUBE1* | 45 | 2.55±0.53 | 3.43±1.0 | **0.04** | 67 | 12.5 | **0.03** |
| 22q13.31 | 43,771,998-43,893,933 | Gain | 121.9 | *MPPED1* | 45 | 2.55±0.53 | 3.43±1.0 | **0.04** | 67 | 12.5 | **0.03** |
| 22q13.32 | 47,208,708-47,252,209 | Gain | 43.5 | *TBC1D22A* | 45 | 2.55±0.53 | 3.43±1.0 | **0.04** | 67 | 12.5 | **0.03** |
| 22q13.32 | 47,261,745-47,270,520 | Gain | 8.8 | *TBC1D22A* | 45 | 2.55±0.53 | 3.43±1.0 | **0.04** | 67 | 12.5 | **0.03** |
| 22q13.32 | 47,288,391-47,401,631 | Gain | 113.2 | *TBC1D22A* | 45 | 2.55±0.53 | 3.43±1.0 | **0.04** | 67 | 12.5 | **0.03** |
| 22q13.32 | 47,401,631-47,487,214 | Gain | 85.6 | *TBC1D22A* | 50 | 2.52±0.51 | 3.57±1.0 | **0.007** | 75 | 12.5 | **0.02** |
| Xp22.33 | 1,465,498-1,499,832 | Loss | 34.3 | ***IL3RA*** | 50 | 3.47±1.01 | 2.6±0.69 | **0.03** | 33 | 75 | 0.17 |
| Xp22.33 | 1,499,832-1,559,835 | Loss | 60 | ***IL3RA****, SLC25A6* | 45 | 3.47±1.01 | 2.6±0.69 | **0.03** | 33 | 62.5 | 0.36 |

*Cancer related genes are in bold while genes related to pancreas are underlined. **^§^** Results expressed as mean tumor size ± one standard deviation (SD)
